# Lipid levels are inversely associated with infectious and all-cause mortality: international MONDO study results^1^[S]

**DOI:** 10.1194/jlr.P084277

**Published:** 2018-06-12

**Authors:** George A. Kaysen, Xiaoling Ye, Jochen G. Raimann, Yuedong Wang, Alice Topping, Len A. Usvyat, Stefano Stuard, Bernard Canaud, Frank M. van der Sande, Jeroen P. Kooman, Peter Kotanko, on behalf of the Monitoring Dialysis Outcomes (MONDO) Initiative

**Affiliations:** Department of Medicine, Division of Nephrology, and Biochemistry and Molecular Medicine, University of California Davis School of Medicine,* Davis, CA; Research Division, Renal Research Institute,† New York, NY; Department of Statistics and Applied Probability, University of California–Santa Barbara,§ Santa Barbara, CA; Fresenius Medical Care North America,** Waltham, MA; Fresenius Medical Care,†† Bad Homburg, Germany; Department of Internal Medicine, Division of Nephrology, Maastricht University Medical Center,§§ Maastricht, The Netherlands; Icahn School of Medicine at Mount Sinai Health System,*** New York, NY

**Keywords:** hemodialysis, infection, innate immune system, high-density lipoprotein cholesterol, low-density lipoprotein cholesterol, triglyceride, endotoxin, Monitoring Dialysis Outcomes

## Abstract

Cardiovascular (CV) events are increased 36-fold in patients with end-stage renal disease. However, randomized controlled trials to lower LDL cholesterol (LDL-C) and serum total cholesterol (TC) have not shown significant mortality improvements. An inverse association of TC and LDL-C with all-cause and CV mortality has been observed in patients on chronic dialysis. Lipoproteins also may protect against infectious diseases. We used data from 37,250 patients in the international Monitoring Dialysis Outcomes (MONDO) database to evaluate the association between lipids and infection-related or CV mortality. The study began on the first day of lipid measurement and continued for up to 4 years. We applied Cox proportional models with time-varying covariates to study associations of LDL-C, HDL cholesterol (HDL-C), and triglycerides (TGs) with all-cause, CV, infectious, and other causes of death. Overall, 6,147 patients died (19.2% from CV, 13.2% from infection, and 67.6% from other causes). After multivariable adjustment, higher LDL-C, HDL-C, and TGs were independently associated with lower all-cause death risk. Neither LDL-C nor TGs were associated with CV death, and HDL-C was associated with lower CV risk. Higher LDL-C and HDL-C were associated with a lower risk of death from infection or other non-CV causes. LDL-C was associated with reduced all-cause and infectious, but not CV mortality, which resulted in the inverse association with all-cause mortality.

Mortality is significantly higher among patients with advanced kidney disease compared with the general population (1). The two leading causes of death in dialysis patients are CVD and infectious diseases (2, 3). CVD is increased 35-fold in patients with stage 5 chronic kidney disease (CKD) (1), and CVD mortality is not reduced upon initiation of dialysis therapy. Although CVD mortality has been declining, the rate of infectious hospitalizations and deaths has been increasing (4). In observational studies, higher total cholesterol and LDL cholesterol (LDL-C) are associated with better survival in chronic hemodialysis (HD) and peritoneal dialysis patients (5–9). Although treatment of hyperlipidemia or dyslipidemia has a modest effect on CVD outcomes in patients with CKD, neither overall mortality nor CVD-related events are significantly affected by lipid-lowering therapy among HD patients (10, 11). If indeed LDL-C has no clear effect on CVD outcomes and is associated with reduced risk of death, the question of the relationship between LDL-C and other outcomes may provide insight into potential mechanisms of protection.

The second leading cause of death among HD patients is infection (4). Epidemiologically, lipoprotein levels have been associated with protection from infectious events (12, 13). HDL cholesterol (HDL-C) plays a role in the transfer of lipotoxins, endotoxin, and lipopolysaccharide (LPS) to the liver, where it is degraded (14–16). HDL-C contains LPS binding protein (LBP) that participates in this transfer (17). Inflammation even in the absence of clinical infection is strongly associated with all-cause and CVD mortality in patients with end-stage renal disease (18). HDL has been shown to be protective against polymicrobial sepsis in mice (19). LDL-C has been shown to be inversely associated with infectious outcomes in patient populations with normal renal function (12) and likely plays a key role in host defense against both bacterial and some viral pathogens (13). Interventional studies in animal models strongly suggest that lipoproteins may potentially play an important role in the innate immune response (20–22) and protect against sepsis in experimental animal models (19). Additionally, infection per se may alter the structure and function of lipoproteins (22, 23). Thus, it is plausible that the observed association of high total cholesterol and LDL-C levels with survival (5–7) and the lack of an effect of lipid-lowering therapies on all-cause mortality in interventional studies (11) is based at least in part upon a protective effect of lipoprotein classes on infectious outcomes.

Some lipoproteins also behave as acute-phase proteins in response to inflammation (24–26), so that it is important to distinguish between lipoprotein levels prior to an infectious event as opposed to their levels during infection, as well as to control for variables associated with inflammation to try to separate cause from effect. We conducted a retrospective cohort study to analysis the outcomes in an international cohort of in-center HD patients from the Monitoring Dialysis Outcomes (MONDO) database (27) to study the relationship between lipid levels during the prior 4-year period and all-cause mortality, death attributed to infectious diseases, and death attributed to cardiovascular (CV), and in order to establish whether any protective effects against infectious death by specific lipoprotein classes offset potential injurious effects on CVD mortality. We also investigated the relationship between each of the lipoprotein classes and noninfectious and non-CV mortality (other causes of mortality) to determine whether the entire effect of specific lipoprotein classes were the result of CV-related mortality, infection-related mortality, or other causes.

## MATERIALS AND METHODS

### Data source

The MONDO initiative is an international retrospective cohort study that comprised all the chronic HD patients from 41 countries of Fresenius Medical Care (FMC) Europe, Middle East, and Africa; FMC South America; FMC Asia Pacific; and United States–Renal Research Institute (RRI); FMC Latin America; Maastricht University Hospital and University of Einthoven in the Netherlands; Hadassah Medical Center in Israel; Imperial College in the United Kingdom; National Heart Institute of Mexico City in Mexico; Nephro Solution and Kuratorium für Dialyze und Nierentransplantation in Germany; and Pontifical Catholic University of Parana in Brazil (27). Corresponding data providers were responsible for the primary data collection and safeguarding the usage of patient data in accordance with local data protection laws and privacy. Patients were stratified into regions per the United Nations geographical scheme (28). All the identifiable variables were removed before data were transferred to FMC-RRI (New York, NY). Multiple levels of internal data validation controls were applied before the data were integrated into a master database. All the research conducted by the MONDO Initiative complied with national and international ethical, compliance, and legal standards. The study was approved by the Western Institutional Review Board (ES-16-005).

### Patient selections and study design

**Fig. 1.**
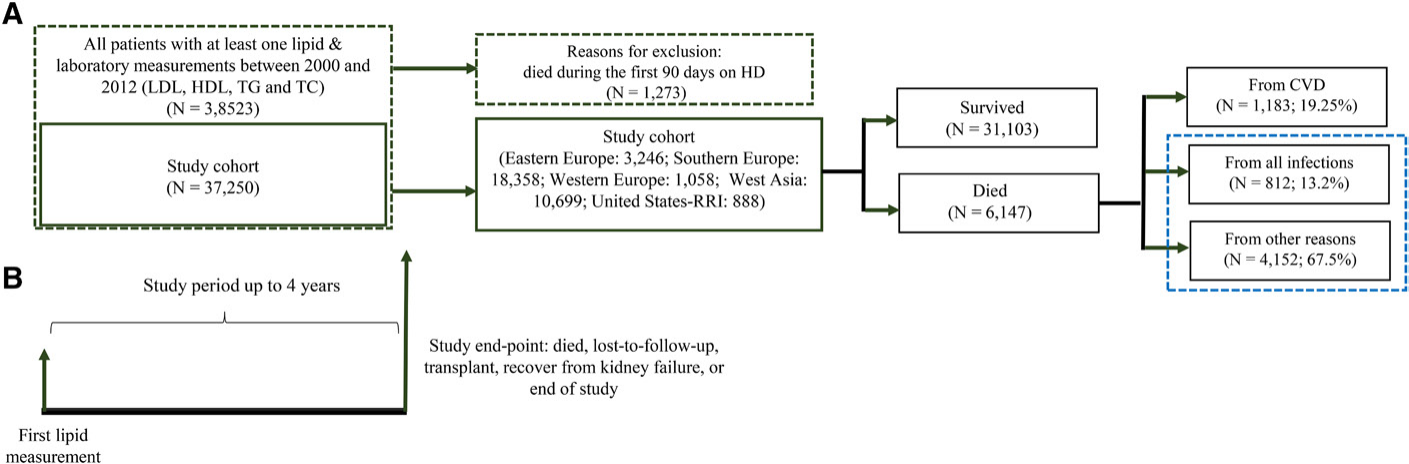
Study population, study flow chart, and study design. TC, total cholesterol.

All the adult patients who survived more than 90 days on HD and with at least one complete lipid panel [LDL-C, HDL-C, and triglycerides (TGs)] measured between January 1, 2000 and December 31, 2012 within the MONDO master database were included. Therefore, the final study population consisted of 37,250 patients from Eastern, Southern, and Western Europe, as well as West Asia and the RRI (Fig. 1A). Patients were included at the first available lipoprotein measurement and were followed for up to 4 years until an event (death, censored, loss-to-follow-up, or recovered from renal failure) occurred (Fig. 1B).

### Exposures and outcomes

The primary exposures of interest were time-varying serum lipid levels of calculated LDL-C, HDL-C, and TG. Given the fact that albumin, creatinine, C-reactive protein (CRP), and neutrophil-to-lymphocyte ratio (NLR), BMI, age, and dialysis vintage (time after initial start of renal replacement therapy) also measured routinely in majority of the study cohort, each of the parameters of interest was treated as a time-dependent parameter while performing the analyses. The primary outcomes of interest were time to all-cause and infectious-based hospitalizations that resulted in mortality (infection-related mortalities; see supplemental data). Secondary outcomes of interest were CV-based hospitalizations that resulted in mortality (CV-related mortality; see supplemental data) and other mortalities that were not infection-related and not CV-related causes.

CV-based hospitalizations were chosen to report arterial injury similar to outcomes chosen in the SHARP trial (11). We considered died due to infection-related or CV-related if the primary International Classification of Diseases (ICD), 9th revision, Clinical Modification (for RRI’s patients) or ICD, 10th revision (for Europe and West Asia’s patients) diagnosis code was related to infection-related or CV-related hospitalizations. The corresponding documented free-text descriptions were applied to further validate the cause-specific mortalities.

### Statistical analyses

Continuous variables were reported as means (± SD) or median (interquartile range) depending on the distribution of the data. To test the significant differences of the continuous variables, Kruskal-Wallis, one-way ANOVA, or ANOVA were performed. Categorical variables were presented as proportions and test via chi-square tests.

Cox proportional hazard regression models with time-varying covariates (29) were performed to examine the univariate associations between each of the lipoproteins (LDL-C, HDL-C, and TGs per mmol/l) and all-cause mortality, infection-related mortality, and CV-related mortality as well as other mortalities in separate models. Up to 48 time-varying values of each lipoprotein per participant using lipoprotein values measured at different time points were included in each analysis. For patients with more than one lipid measurement during the baseline period, the previous value was replaced by the upcoming value while conducting the analyses. To account for changes in serum lipid levels over time and assess the associations between lipid levels and each of the cause-specific deaths, the following six models with incremental levels of adjustments were conducted: *1*) model 1: each serum lipoprotein (noted as continuous value) with age, dialysis vintage, gender, vascular access, and BMI, as well as the geographical region that the patients came from; *2*) model 2: model 1 plus diabetic status; *3*) model 3: model 2 plus serum creatinine levels; *4*) model 4: model 3 plus serum albumin; *5*) model 5: model 3 plus serum CRP; and *6*) model 6: model 3 plus serum albumin, creatinine, CRP, and NLR levels. We defined model 6 as the preferable model, which contained nutritional and inflammation markers, because we hypothesized that the inverse association between HDL-C, LDL-C, and infection-related mortality should remain while accounting for the effect of nutritional status and inflammation.

Analyses were performed with SAS (Version 9.4; Cary, NC) and R (Version 3.1.3) (30).

### Clinical and laboratory analyses

Clinical and laboratory data were directly imported electronically in all the European, West Asian, and all North America-RRI clinics. All the lipoproteins were measured routinely from monthly to half-yearly in Europe and West Asia clinics, measured annually in FMC-RRI clinics. Serum albumin was measured by the bromocresol green method in most of the studied clinics. In Portugal, bromocresol purple method was used. Both methods were calibrated to international standard European Reference Materials DA 470k/International Federation of Clinical Chemistry and Laboratory Medicine. Serum creatinine was measured routinely by the Jaffe method in all FMC Europe and RRI clinics. Serum CRP level was measured with conventional assay in all the studied clinics. NLR were measured routinely monthly in all the regions. All the clinical and laboratory parameters that were measured by different methods were calibrated to US standards before any analyses were conducted (31).

## RESULTS

In total, 37,250 HD patients were included in this multinational cohort, with median age of 64 years; 59% male; 36% entered the study cohort as incident patients; median dialysis vintage (time on dialysis) was 3.04 years at the beginning of the study; median follow-up time (time from first lipid measurement to event) was 3.4 years; 6,246 (16.77%) from Eastern Europe, 18,358 (49.28%) from Southern Europe; 1,058 (2.84%) from Western Europe; 10,699 (28.72%) from West Asia; and 888 (2.38%) from United States-RRI. Patient characteristics stratified by cause-specific death are presented in **Table 1**. The flow chart of the study cohort and study design is presented in **Fig. 1A**. Out of 37,250 patients included in the study, 6,147 died due to all causes, with the rate of 163 per 1,000 patient years; 1,183 (19.24%) died due to CV, 883 (13.21%) died due to infections, 4,024 died due to any other causes that are not CV and not infections, and 57 (0.9%) had missing information of cause of death (Fig. 1A).

**TABLE 1.** Baseline characteristics of the study cohort

Parameters	All Patients	Died from All Reasons	Died from CV	Died from All Infections	Died from Others (Not Infections and Not CV)	Patients Who Survived
No. of patients	37,250	6,147	1,183	812	4,152	31,103
Age (years), Median (Q1, Q3)	64 (52, 74)	72 (63, 79)	71 (62, 78)	72 (63, 79)	73 (63, 79)	62 (51, 73)
BMI	25.71 ± 6.62	24.27 ± 5.48	24.74 ± 5.06	24.59 ± 5.54	24.06 ± 5.58	26.72 ± 5.57
Diabetics (%)	15.57	18.50	17.95	21.43	18.08	15.00
Gender: male (%)	58.60	59.77	62.55	57.64	59.39	58.37
Catheter (%)	44.68	47.37	50.04	47.04	46.68	44.14
Vintage (years), Median (Q1, Q3)	3.04 (0.13, 4.15)	2.63 (0.11, 3.78)	2.73 (0.11, 4.00)	2.51 (0.10, 3.41)	2.64 (0.11, 3.74)	3.12 (0.13, 4.24)
Serum albumin (g/dl)	3.85 ± 0.42	3.68 ± 0.49	3.75 ± 0.45	3.62 ± 0.52	3.67 ± 0.49	3.89 ± 0.41
Serum creatinine ([mg/dl)	7.74 ± 2.35	6.77 ± 2.09	6.96 ± 2.07	6.74 ± 2.08	6.72 ± 2.09	7.94 ± 0.36
CRP (mg/dl), Median (Q1, Q3)	8.73 (1.16, 10.09)	12.64 (1.83, 16.00)	11.29 (1.53, 15.07)	14.76 (2.66, 19.34)	12.61 (1.82, 15.72)	7.96 (1.08, 1.24)
NLR	3.22 ± 2.47	3.75 ± 3.00	3.52 ± 2.05	4.00 ± 3.40	3.77 ± 3.11	3.12 ± 2.34
**LDL-C (mmol/l)**						
All regions	2.45 ± 0.87	2.39 ± 0.88	2.46 ± 0.92	2.30 ± 0.91	2.39 ± 0.87	2.47 ± 0.87
Eastern Europe (n = 6,246)	2.64 ± 1.02	2.54 ± 0.97	2.57 ± 1.03	2.51 ± 1.00	2.53 ± 0.95	2.66 ± 1.03
Southern Europe (n = 18,358)	2.27 ± 0.78	2.21 ± 0.81	2.20 ± 0.83	2.18 ± 0.83	2.22 ± 0.81	2.29 ± 0.77
Western Europe (n = 1,058)	2.31 ± 0.93	2.31 ± 0.93	2.20 ± 0.83	2.05 ± 1.63	2.35 ± 0.92	2.31 ± 0.93
West Asia (n = 10,699)	2.71 ± 0.83	2.70 ± 0.87	2.84 ± 0.85	2.65 ± 1.03	2.67 ± 0.85	2.71 ± 0.83
United States (n = 888)	2.03 ± 0.82	2.08 ± 0.81	1.96 ± 0.87	2.15 ± 0.71	2.07 ± 0.82	2.02 ± 0.82
HDL-C (mmol/l)						
All regions	1.05 ± 0.34	1.03 ± 0.34	1.01 ± 0.32	1.07 ± 0.34	1.03 ± 0.34	1.06 ± 0.34
Eastern Europe (n = 6,246)	1.06 ± 0.36	1.00 ± 0.39	1.06 ± 0.39	1.04 ± 0.38	0.98 ± 0.38	1.07 ± 0.36
Southern Europe (n = 18,358)	1.14 ± 0.34	1.11 ± 0.33	1.09 ± 0.34	1.14 ± 0.33	1.11 ± 0.33	1.15 ± 0.34
Western Europe (n = 1,058)	1.90 ± 0.41	1.12 ± 0.35	1.14 ± 0.41	1.32 ± 0.19	1.11 ± 0.34	1.20 ± 0.42
West Asia (n = 10,699)	0.89 ± 0.26	0.88 ± 0.27	0.87 ± 0.24	0.84 ± 0.25	0.89 ± 0.27	0.90 ± 0.25
United States (n = 888)	1.09 ± 0.37	1.10 ± 0.38	1.00 ± 0.31	1.06 ± 0.30	1.11 ± 0.40	1.09 ± 0.36
TG (mmol/l)						
All regions	1.86 ± 1.02	1.72 ± 0.95	1.80 ± 1.01	1.71 ± 0.95	1.69 ± 0.93	1.89 ± 1.02
Eastern Europe (n = 6,246)	1.92 ± 1.13	1.87 ± 1.10	1.81 ± 1.13	1.89 ± 0.94	1.89 ± 1.11	1.93 ± 1.14
Southern Europe (n = 18,358)	1.74 ± 0.88	1.57 ± 0.78	1.63 ± 0.82	1.59 ± 0.78	1.55 ± 0.77	1.77 ± 0.90
Western Europe (n = 1,058)	1.63 ± 0.90	1.42 ± 0.68	1.50 ± 0.78	1.17 ± 0.71	1.41 ± 0.66	1.65 ± 0.91
West Asia (n = 10,699)	2.07 ± 1.13	1.93 ± 1.09	2.06 ± 1.18	1.98 ± 1.28	1.88 ± 1.04	2.10 ± 1.14
United States (n = 888)	1.84 ± 1.07	1.78 ± 1.09	1.71 ± 0.75	2.04 ± 1.34	1.73 ± 1.04	1.86 ± 1.07

Data are presented as mean ± SD for normal distributed variables, or median for not normal distributed variables (25th and 75th percentile). All the categorical variables were reported at the time of first lipid measurement. Contentious variables were presented as the average value during the study period.

### All-cause mortality

**Fig. 2.**
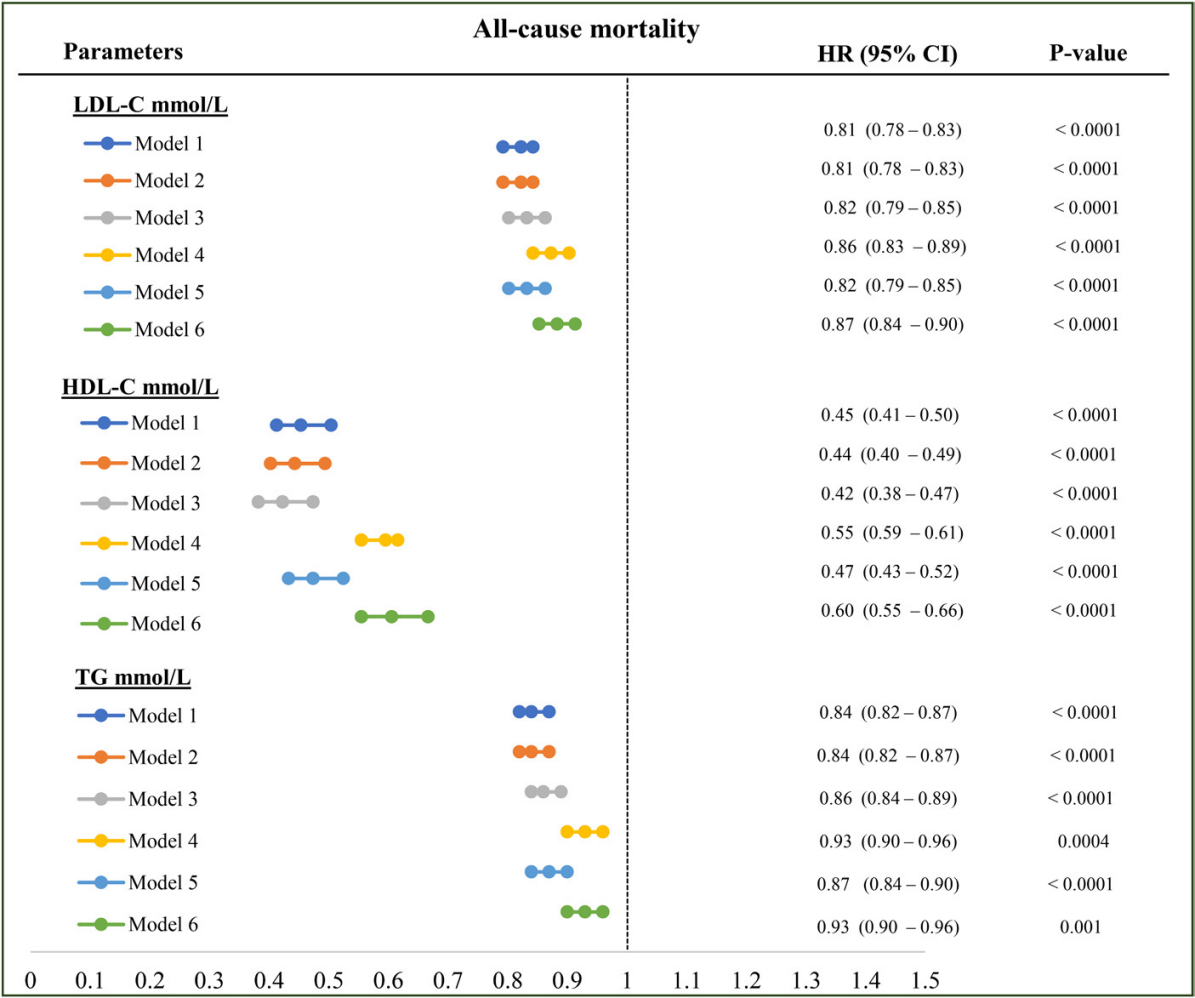
Association between each lipoprotein and all-cause mortality. Model 1: LDL-C or HDL-C or TG plus age, vintage, gender, vascular access, BMI, regions; model 2: model 1 plus diabetes; model 3: model 2 plus serum creatinine level; model 4: model 3 plus serum albumin level; model 5: model 3 plus serum CRP; model 6: model 3 plus serum albumin, CRP, and NLR level. All the analyses were performed by Cox proportional model with time-varying covariates. HRs are reported as per mmol/l.

By univariate analyses, LDL-C [hazard ratio (HR): 0.85, 95% CI: 0.82–0.87], HDL-C (HR: 0.64, 95% CI: 0.59–0.79), and TG (HR: 0.77, 95% CI: 0.75–0.79) levels were positively associated with survival in HD patients (**Table 2**). By multivariate analysis, higher LDL-C (HR: 0.82, 95% CI: 0.79–0.85), HDL-C (HR: 0.42, 95% CI: 0.38–0.47), and TG (HR: 0.86, 95% CI: 0.84–0.89) concentration were significantly associated with lower all-cause mortality after adjustment for demographics and diabetic status, as well as following adjustment for nutritional marker, noted as serum creatinine (**Fig. 2**, model 3). Higher LDL-C, HDL-C, and TGs remain significantly associated with lower all-cause mortality while adding serum albumin (Fig. 2, model 4) or CRP (Fig. 2, model 5) into the model. In the fully adjusted model (Fig. 2, model 6) LDL-C (HR: 0.87, 95% CI: 0.84–0.90), HDL-C (HR: 60, 95% CI: 0.55–0.66), and TGs (HR: 0.93, 95% CI: 0.90–0.96) (Fig. 2, model 6) remained associated with lower mortality, suggesting that the effects of the lipoproteins were not mediated by nutritional or inflammatory status.

**TABLE 2.** Association between time-varying serum lipid levels and outcomes without adjustments

Lipid Parameters	All-Cause Mortality		CV-Related Mortality		Infection-Related Mortality		Other Mortality (Not Infection and CV Related)	
	HR (95% CI)	*P*	HR (95% CI)	*P*	HR (95% CI)	*P*	HR (95% CI)	*P*
LDL (mmol/l)	0.87 (0.84–0.89)	<0.0001	0.97 (0.92–1.03)	0.32	0.78 (0.72–0.84)	<0.0001	0.84 (0.82–0.87)	<0.0001
HDL (mmol/l)	0.68 (0.64–0.74)	<0.0001	0.61 (0.52–0.73)	<0.0001	0.89 (0.74–1.07)	0.22	0.70 (0.65–0.76)	<0.0001
TG (mmol/l)	0.79 (0.77–0.82)	<0.0001	0.90 (0.85–0.95)	0.0001	0.77 (0.71–0.83)	<0.0001	0.77 (0.74–0.79)	<0.0001

### CVD-related mortality

**Fig. 3.**
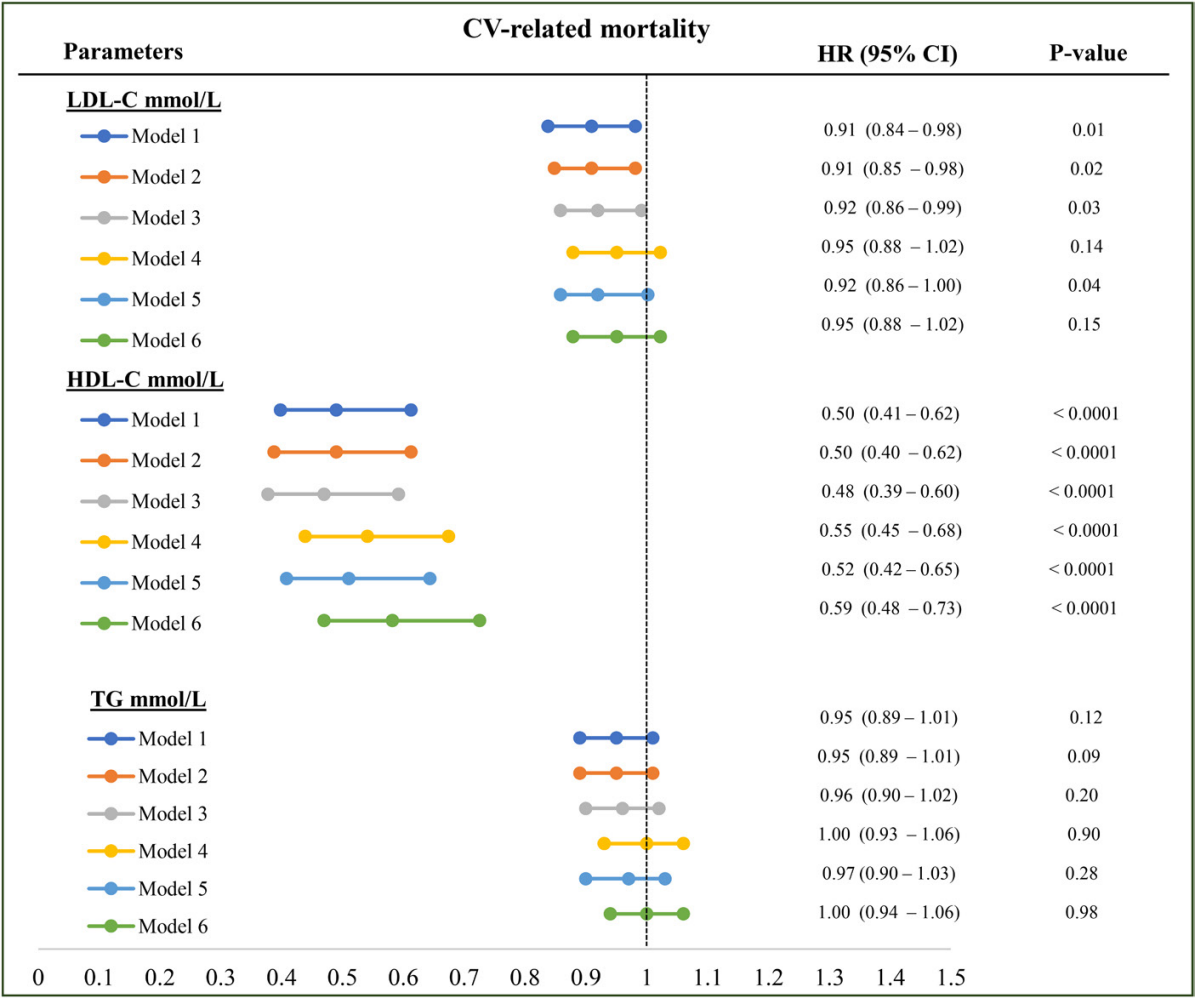
Association between each lipoprotein and CV-related mortality. Model 1: LDL-C or HDL-C or TG plus age, vintage, gender, vascular access, BMI, regions; model 2: model 1 plus diabetes; model 3: model 2 plus serum creatinine level; model 4: model 3 plus serum albumin level; model 5: model 3 plus serum CRP; model 6: model 3 plus serum albumin, CRP, and NLR level. All the analyses were performed by Cox proportional model with time-varying covariates. HRs are reported as per mmol/l.

We found no statistically significant associations between LDL-C and CV mortality despite adjustment for demographics, nutritional or inflammatory surrogates (HR 0.95, 95% CI 0.88–1.02, model 6) (Table 2 and **Fig. 3**). HDL-C levels were significantly associated with reduced CV mortality in all models (Table 2 and Fig. 3). TG was significantly associated with reduced CV mortality (HR: 0.89, 95% CI: 0.84–0.94, Table 2) in the univariate analyses, but lost its significance when further adjusted for demographics, malnutrition, and inflammation (Fig. 3, models 1–6).

### Infection-related mortality

**Fig. 4.**
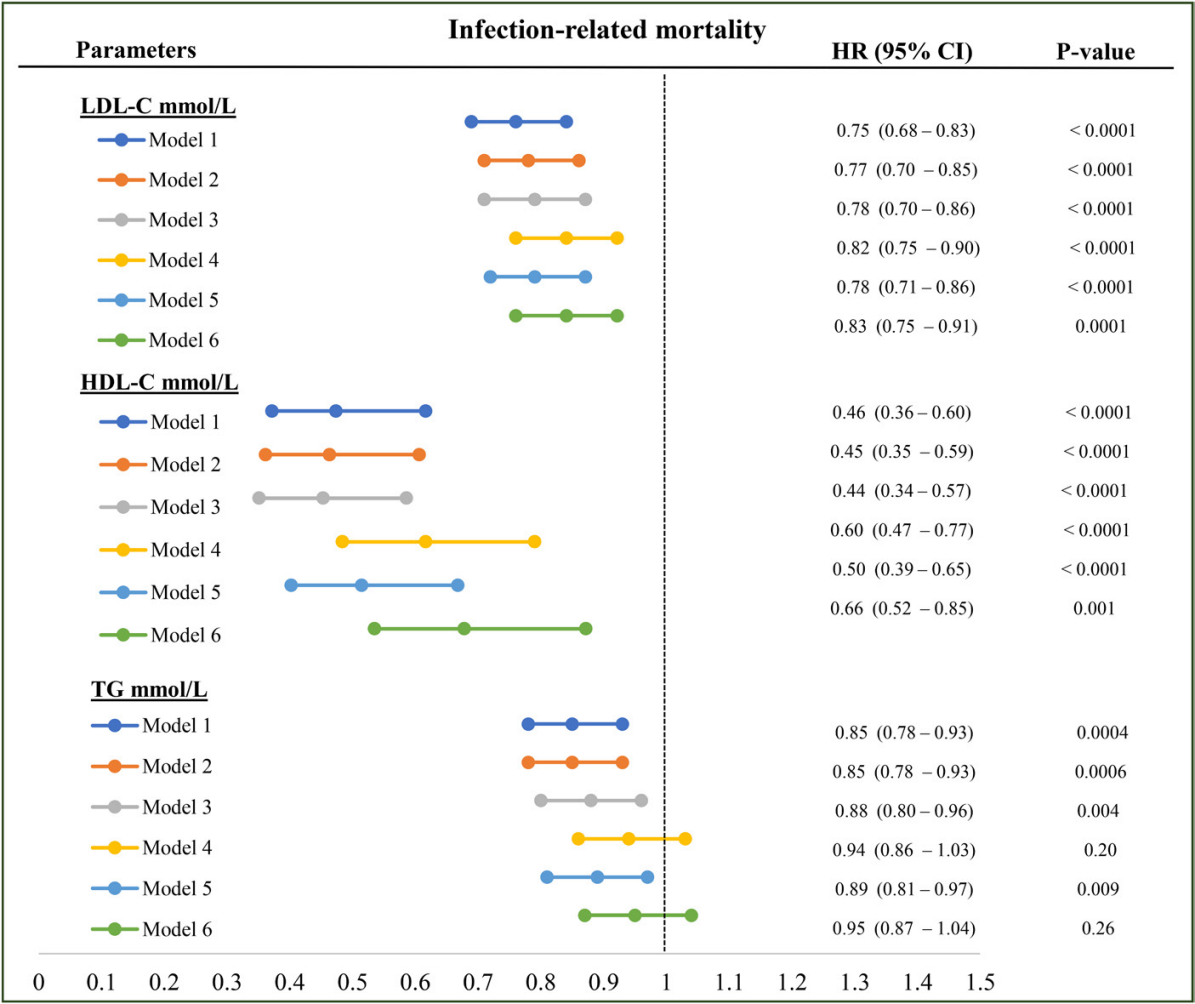
Association between each lipoprotein and infection-related mortality. Model 1: LDL-C or HDL-C or TG plus age, vintage, gender, vascular access, BMI, regions; model 2: model 1 plus diabetes; model 3: model 2 plus serum creatinine level; model 4: model 3 plus serum albumin level; model 5: model 3 plus serum CRP; model 6: model 3 plus serum albumin, CRP, and NLR level. All the analyses were performed by Cox proportional model with time-varying covariates. HRs are reported as per mmol/l.

Higher LDL-C was significantly associated with lower infection-related mortality in the univariate model (HR: 0.76; 95% CI: 0.70–0.82). The inverse associations between LDL-C and infection-related mortality remained statistically significant after adjustment for demographics (HR: 0.75; 95% CI: 0.68–0.83; **Fig. 4**, model 1), demographics plus diabetic status (HR: 0.77; 95% CI: 0.70–0.86; Fig. 4, model 2), demographics, plus diabetes, creatinine (HR; 0.78; 95% CI: 0.70–0.86; Fig. 4, model 3), and in the fully adjusted model (HR: 0.83; 95% CI: 0.75–0.91; Fig. 4, model 6), although there was some attenuation in the protective effect after adjusting for creatinine and albumin. HDL-C was protective against infectious mortality in univariate and multivariate analyses (Table 2 and Fig. 4) independent of both nutritional and inflammatory biomarkers. TGs were also inversely associated with infectious mortality in the univariate model (HR: 0.75; 95% CI: 0.69–0.81; Table 2), as well as in the models adjusted for demographics, diabetes, serum creatinine, and CRP (Fig. 4, models 1–3 and 5) but not following adjustment for serum albumin (Fig. 4, models 4 and 6).

### Other (not infection-related and not CVD-related) mortality

**Fig. 5.**
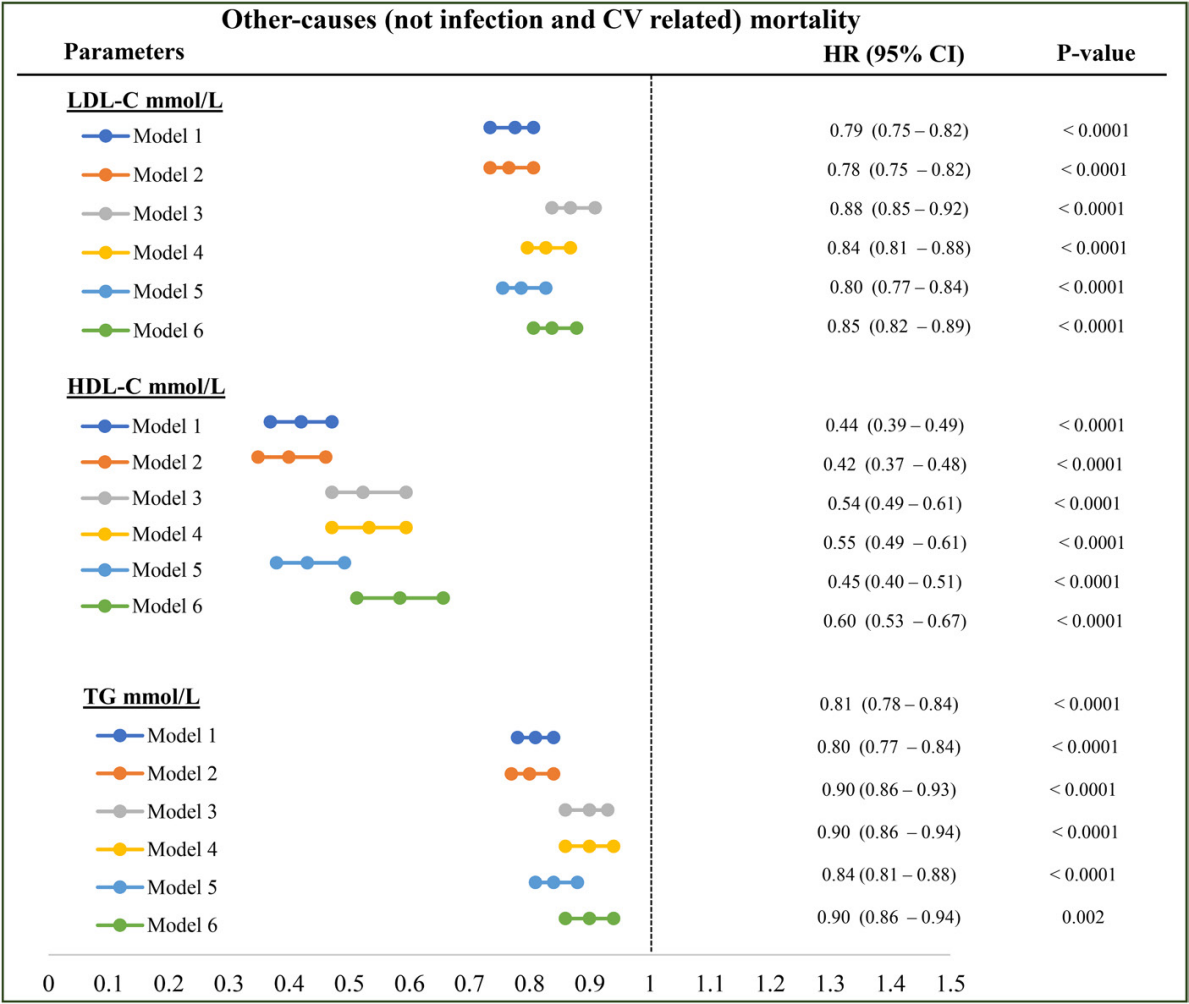
Association between each lipoprotein and other (not infection-related and not CV-related) mortality. Model 1: LDL-C or HDL-C or TG plus age, vintage, gender, vascular access, BMI, regions; model 2: model 1 plus diabetes; model 3: model 2 plus serum creatinine level; model 4: model 3 plus serum albumin level; model 5: model 3 plus serum CRP; model 6: model 3 plus serum albumin, CRP, and NLR level. All the analyses were performed by Cox proportional model with time-varying covariates. HRs are reported as per mmol/l.

All the lipoproteins (LDL-C, HDL-C, and TGs) were significantly associated with reduced noninfectious and non-CV-related mortality, both in univariate analyses (Table 2) and multivariate analyses with incremental adjustments (**Fig. 5**).

## DISCUSSION

Mortality is inversely associated with total cholesterol and LDL-C cholesterol in the dialysis population (7, 32) in contrast to the general population (33). In this present study, we also found higher LDL-C, HDL-C, and TG levels measured over time were significantly associated with better survival. Additionally, we did not observe any association between LDL-C level or TG and CV mortality, even after adjustment for nutritional surrogate biomarkers or the inflammatory biomarkers CRP and NLR, whereas HDL-C is protective. Because the association between cholesterol levels and mortality in the general population is primarily a consequence of vascular disease (34), our observations that LDL-C levels have no association with CV outcomes partially explain the difference between the dialysis population and general population. Part of the residual protective effect of lipoproteins, accounting for their association with reduced mortality, appears to be a consequent part of reduced infectious mortality, because the second leading cause of death is infections (2, 35). Additionally, all of the lipoprotein classes were associated with reduction in other causes of mortality that were not categorized as infectious or CV mortality.

The finding of reduced infectious mortality may be linked to known protective effects both of HDL-C and of other lipoprotein classes. Infection has been shown to affect the structure of lipoproteins (22, 23) and lipoprotein structure independent of lipoprotein concentration has also been shown to be associated with infectious risk (36). The lack of an association between lipids, and specifically LDL-C and CV outcomes, may be that non-lipoprotein-related processes associated with inflammation (18, 37, 38) or calcium and phosphorous metabolism (39, 40) that play a more limited role in the general population are responsible for the large increase in CVD outcomes (1) and dominate CVD outcomes in dialysis patients, obscuring lipoprotein-related pathways of injury. Analysis of patients enrolled in the CHOICE study found that total cholesterol increased CVD risk if only subjects who were not inflamed were included in the analysis (37, 38); nevertheless, in that study, the mortality risk for uninflamed patients with high total cholesterol was significantly less than that for inflamed patients at any level of cholesterol. We found no effect of LDL-C, even after adjusting for inflammatory processes, both in this population as well as in a smaller prospective study in which LDL-C was measured directly (41), although infectious hospitalizations rather than infectious mortality were the primary outcome in that study. The question raised then now is not why LDL-C levels do not increase mortality risk, but what is the mechanism whereby LDL-C is protective in this population?

It has been observed that among patients with normal renal function, both LDL-C and other apo B-associated lipoproteins are associated with lower risk of infection (42) and improved survival among patients with sepsis (43). However, many of these observations were based on lipid levels at the time of hospitalization for septic events, which might be confounded by severity of inflammation, because greater inflammation is expected to reduce lipid levels more (24, 26, 44, 45). Our observations here are strengthened by the predictive effect of measurements carried out at a time distant from the outcomes, as much as 4 years prior to any event noted. Furthermore, in longitudinal analysis of the relationship between apoB and apo AI and their ratio with inflammatory events measured as CRP, we previously reported no temporal variability of apo B associated with changes in CRP, whereas apo A I varied reciprocally with CRP (24).

The observation that infectious mortality is inversely associated with both LDL-C and HDL-C levels, even after adjusting for nutritional and inflammatory markers, is consistent with prior observations by us (41) and consistent with reported protective effect of LDL-C and other lipoproteins from infection or bacterial toxins (19, 20) and protection against the lethal effect of injected LPS in animal models (46, 47). Lipoproteins interact with bacterial toxins, many of which are lipids, providing a potential biological link between epidemiologic observations and mechanism (48–53). In addition, LDL-C absorbs exotoxins secreted by Gram-positive bacteria (47). The presence of increased inflammation (18) and endogenous endotoxin exposure in dialysis patients (54, 55) would be expected both to increase CV risk from inflammation and change the overall effect of lipoproteins, specifically of LDL-C, to favor protection, as observed here.

HDL-C contains LBP (17), an acute phase protein that is in part carried by HDL-C and plays a role in neutralizing the biological effect of LPS (56), in part by transferring LPS from HDL-C to lipoproteins targeted by the hepatic LDL-C receptor (57) for subsequent hepatic uptake and disposal (58). This could help to support the explanation of the protective effect that HDL-C played in the cause of death due to infections. The effect of TG-rich lipoproteins may be more difficult to estimate from these data because VLDLs, chylomicrons, and remnant particles were not measured directly. However, we also found that LDL-C and HDL-C are associated with a reduced rate of mortality from other residual causes of death. This result is similar to what is observed in the general population for total cholesterol (34). Unlike the statistically significant reduction in infectious death, there is no obvious mechanism to explain the protective effect of LDL-C and HDL-C against residual causes of death, and one weakness of this study is that we did not further analyze the distribution of deaths not associated with CV or infectious hospitalizations. Many of these deaths occurred outside of the hospital. It remains possible that residual nutritional factors contributed, although we have controlled for BMI as well as albumin and creatinine. Cancer represents approximately 7% of all-cause mortality in a study of European HD patients (59). There may also be a protective effect of total cholesterol and cancer outcomes in some forms of cancer (60), although there may be confounding effects of nutritional intake as well (34); however, the relationship between cancer death, and specifically cancer type and lipids and outcomes in HD patients is beyond the scope of this investigation. Cachexia and withdrawal from dialysis each represent approximately 5% from the same data base and would be expected to be associated with reduced levels of nutritional biomarkers and decreased lipid levels (47). A variety of nutritional measures are associated with increased overall mortality risk in HD patients (61, 62).

These observations should also be viewed in the context that LDL-C was not measured directly nor fasting; however, the lack of a relationship between LDL-C levels and CV outcomes is consistent with the failure of interventional trials to alter CVD risk in HD patients (10, 11). Our CV rate is lower than reported by the U.S. Renal Data System (4); however, we focused our CV classification to represent arterial injury rather than all CV events that may be due to volume overload or electrolyte abnormalities. Although the majority of our study cohort is from Europe, the event rate is similar to those reported in a European population focusing on the same outcomes (59).

One of the strengths of current study is that the sample size is relatively large, and the sample was diverse according to geographical regions. This allowed us to study regions that routinely perform lipid panels as well as CRP measurement without indication reducing a selection bias in sampling. Most of the study cohort is from Europe, which contains more than 95% white patients, which may limit the applicability of these finding to other racial groups. Although the current study is a retrospective observational study, we have very limited documentation of medication to include in this analysis in the current study. Another limitation of the current study is that we do not have enough documentation of comorbid conditions in the current subset study cohort. Furthermore, a number of additional nutritional related biomarkers—Subjective Global Assessment, muscle mass, skin fold thickness—are unavailable. Thus, residual confounding factors still cannot be eliminated.

## Supplementary Material

Supplemental Data

## References

[1] Go A. S., Chertow G. M., Fan D., McCulloch C. E., and Hsu C. Y. 2004 Chronic kidney disease and the risks of death, cardiovascular events, and hospitalization. N. Engl. J. Med. 351: 1296–1305. [Erratum, 2008. *N. Engl. J. Med.* 18: 4.]. 15385656 10.1056/NEJMoa041031

[2] U.S. Renal Data System. 2011. USRDS 2011 Annual Data Report: Atlas of Chronic Kidney Disease and End-Stage Renal Disease in the United States. National Institutes of Health, National Institute of Diabetes and Digestive and Kidney Diseases, Bethesda, MD.

[3] Cheng X., Nayyar S., Wang M., Li X., Sun Y., Huang W., Zhang L., Wu H., Jia Q., Liu W., 2013 Mortality rates among prevalent hemodialysis patients in Beijing: a comparison with USRDS data. Nephrol. Dial. Transplant. 28: 724–732. 22907953 10.1093/ndt/gfs326

[4] U.S. Renal Data System. 2013. USRDS 2013 Annual Data Report: Atlas of Chronic Kidney Disease and End-Stage Renal Disease in the United States. National Institutes of Health, National Institute of Diabetes and Digestive and Kidney Diseases, Bethesda, MD.

[5] Goldwasser P., Mittman N., Antignani A., Burrell D., Michel M. A., Collier J., and Avram M. M. 1993 Predictors of mortality in hemodialysis patients. J. Am. Soc. Nephrol. 3: 1613–1622. 8507818 10.1681/ASN.V391613

[6] Avram M. M., Mittman N., Bonomini L., Chattopadhyay J., and Fein P. 1995 Markers for survival in dialysis: a seven-year prospective study. Am. J. Kidney Dis. 26: 209–219. 7611254 10.1016/0272-6386(95)90176-0

[7] Lowrie E. G., Huang W. H., and Lew N. L. 1995 Death risk predictors among peritoneal dialysis and hemodialysis patients: a preliminary comparison. Am. J. Kidney Dis. 26: 220–228. 7611256 10.1016/0272-6386(95)90177-9

[8] Kilpatrick R. D., McAllister C. J., Kovesdy C. P., Derose S. F., Kopple J. D., and Kalantar-Zadeh K. 2007 Association between serum lipids and survival in hemodialysis patients and impact of race. J. Am. Soc. Nephrol. 18: 293–303. 17167113 10.1681/ASN.2006070795

[9] Park C. H., Kang E. W., Park J. T., Han S. H., Yoo T. H., Kang S. W., and Chang T. I. 2017 Association of serum lipid levels over time with survival in incident peritoneal dialysis patients. J. Clin. Lipidol. 11: 945–954.e3. 28669685 10.1016/j.jacl.2017.06.004

[10] Wanner C., Krane V., März W., Olschewski M., Mann J. F., Ruf G., and Ritz E.; German Diabetes and Dialysis Study Investigators. 2005 Atorvastatin in patients with type 2 diabetes mellitus undergoing hemodialysis. N. Engl. J. Med. 353: 238–248. 16034009 10.1056/NEJMoa043545

[11] Baigent C., Landray M. J., Reith C., Emberson J., Wheeler D. C., Tomson C., Wanner C., Krane V., Cass A., Craig J., ; SHARP Investigators. 2011 The effects of lowering LDL cholesterol with simvastatin plus ezetimibe in patients with chronic kidney disease (Study of Heart and Renal Protection): a randomized placebo-controlled trial. Lancet. 377: 2181–2192. 21663949 10.1016/S0140-6736(11)60739-3 PMC3145073

[12] Iribarren C., Jacobs Jr D. R., Sidney S., Claxton A. J., and Feingold K. R. 1998 Cohort study of serum total cholesterol and in-hospital incidence of infectious diseases. Epidemiol. Infect. 121: 335–347. 9825784 10.1017/s0950268898001435 PMC2809530

[13] Feingold K. R., and Grunfeld C. 2012 Lipids: a key player in the battle between the host and microorganisms. J. Lipid Res. 53: 2487–2489. 23075464 10.1194/jlr.E033407 PMC3494250

[14] Level J. H., Marquart J. A., Abraham P. R., van den Ende A. E., Molhuizen H. O., van Deventer S. J., and Meijers J. C. 2005 Lipopolysaccharide is transferred from high-density to low-density lipoproteins by lipopolysaccharide-binding protein and phospholipid transfer protein. Infect. Immun. 73: 2321–2326. 15784577 10.1128/IAI.73.4.2321-2326.2005 PMC1087464

[15] Berbée J. F., Havekes L. M., and Rensen P. C. 2005 Apolipoproteins modulate the inflammatory response to lipopolysaccharide. J. Endotoxin Res. 11: 97–103. 15949136 10.1179/096805105X35215

[16] Read T. E., Harris H. W., Grunfeld C., Feingold K. R., Kane J. P., and Rapp J. H. 1993 The protective effect of serum lipoproteins against bacterial lipopolysaccharide. Eur. Heart J. 14: 125–129. 8131781

[17] Wurfel M. M., Kunitake S. T., Lichenstein H., Kane J. P., and Wright S. D. 1994 Lipopolysaccharide (LPS)-binding protein is carried on lipoproteins and acts as a cofactor in the neutralization of LPS. J. Exp. Med. 180: 1025–1035. 8064223 10.1084/jem.180.3.1025 PMC2191628

[18] Yeun J. Y., Levine R. A., Mantadilok V., and Kaysen G. A. 2000 C-reactive protein predicts all-cause and cardiovascular mortality in hemodialysis patients. Am. J. Kidney Dis. 35: 469–476. 10692273 10.1016/s0272-6386(00)70200-9

[19] Guo L., Ai J., Zheng Z., Howatt D. A., Daugherty A., Huang B., and Li X. A. 2013 High density lipoprotein protects against polymicrobe-induced sepsis in mice. J. Biol. Chem. 288: 17947–17953. 23658016 10.1074/jbc.M112.442699 PMC3689940

[20] Han R. 2010 Plasma lipoproteins are important components of the immune system. Microbiol. Immunol. 54: 246–253. 20377753 10.1111/j.1348-0421.2010.00203.x

[21] Ravnskov U. 2003 High cholesterol may protect against infections and atherosclerosis. QJM. 96: 927–934. 14631060 10.1093/qjmed/hcg150

[22] Khovidhunkit W., Kim M. S., Memon R. A., Shigenaga J. K., Moser A. H., Feingold K. R., and Grunfeld C. 2004 Effects of infection and inflammation on lipid and lipoprotein metabolism: mechanisms and consequences to the host. J. Lipid Res. 45: 1169–1196. 15102878 10.1194/jlr.R300019-JLR200

[23] Kitchens R. L., Thompson P. A., Munford R. S., and O’Keefe G. E. 2003 Acute inflammation and infection maintain circulating phospholipid levels and enhance lipopolysaccharide binding to plasma lipoproteins. J. Lipid Res. 44: 2339–2348. 12923224 10.1194/jlr.M300228-JLR200

[24] Kaysen G. A., Dalrymple L. S., Grimes B., Chertow G. M., Kornak J., and Johansen K. 2014 Changes in serum inflammatory markers are associated with changes in apolipoprotein A1 but not B after the initiation of dialysis. Nephrol. Dial. Transplant. 29: 430–437. 24009290 10.1093/ndt/gft370 PMC3910339

[25] Liao K. P., Playford M. P., Frits M., Coblyn J. S., Iannaccone C., Weinblatt M. E., Shadick N. S., and Mehta N. N. 2015 The association between reduction in inflammation and changes in lipoprotein levels and HDL cholesterol efflux capacity in rheumatoid arthritis. J. Am. Heart Assoc. 4: e001588. 25637346 10.1161/JAHA.114.001588 PMC4345878

[26] Esteve E., Ricart W., and Fernández-Real J. M. 2005 Dyslipidemia and inflammation: an evolutionary conserved mechanism. Clin. Nutr. 24: 16–31. 15681098 10.1016/j.clnu.2004.08.004

[27] von Gersdorff G. D., Usvyat L., Marcelli D., Grassmann A., Marelli C., Etter M., Kooman J. P., Power A., Toffelmire T., Haviv Y. S., 2013 Monitoring dialysis outcomes across the world—the MONDO Global Database Consortium. Blood Purif. 36: 165–172. 24496186 10.1159/000356088

[28] United Nations Statistics Division. Composition of macro geographical (continental) regions, geographical sub-regions, and selected economic and other groupings. Accessed February 1, 2018, at http://unstats.un.org/unsd/methods/m49/m49regin.htm

[29] Fisher L. D., and Lin D. Y. 1999 Time dependent covariates in the Cox proportional-hazards regression model. Annu. Rev. Public Health. 20: 145–157. 10352854 10.1146/annurev.publhealth.20.1.145

[30] R Core Team. 2015. R: a language and environment for statistical computing. R Foundation for Statistical Computing, Vienna, Austria.

[31] Usvyat L. A., Haviv Y. S., Etter M., Kooman J., Marcelli D., Marelli C., Power A., Toffelmire T., Wang Y., and Kotanko P. 2013 The MONitoring Dialysis Outcomes (MONDO) initiative. Blood Purif. 35: 37–48. 23343545 10.1159/000345179

[32] Lowrie E. G., and Lew N. L. 1990 Death risk in hemodialysis patients: the predictive value of commonly measured variables and an evaluation of death rate differences between facilities. Am. J. Kidney Dis. 15: 458–482. 2333868 10.1016/s0272-6386(12)70364-5

[33] Greenland P., Alpert J. S., Beller G. A., Benjamin E. J., Budoff M. J., Fayad Z. A., Foster E., Hlatky M. A., Hodgson J. M., Kushner F. G., ; American College of Cardiology Foundation. 2010 2010 ACCF/AHA guideline for assessment of cardiovascular risk in asymptomatic adults: a report of the American College of Cardiology Foundation/American Heart Association Task Force on Practice Guidelines. J. Am. Coll. Cardiol. 56: e50–e103. 21144964 10.1016/j.jacc.2010.09.001

[34] Jacobs D., Blackburn H., Higgins M., Reed D., Iso H., McMillan G., Neaton J., Nelson J., Potter J., and Rifkind B. 1992 Report of the conference on low blood cholesterol: mortality associations. Circulation. 86: 1046–1060. 1355411 10.1161/01.cir.86.3.1046

[35] Steenkamp R., Rao A., and Fraser S. 2016 UK Renal Registry 18th annual report (December 2015) chapter 5: survival and causes of death in UK adult patients on renal replacement therapy in 2014: national and centre-specific analyses. Nephron. 132(Suppl 1): 111–144. 27115403 10.1159/000444819

[36] Krishnan S., Shimoda M., Sacchi R., Kailemia M. J., Luxardi G., Kaysen G. A., Parikh A. N., Ngassam V. N., Johansen K., Chertow G. M., 2017 HDL glycoprotein composition and site-specific glycosylation differentiates between clinical groups and affects IL-6 secretion in lipopolysaccharide-stimulated monocytes. Sci. Rep. 7: 43728. 28287093 10.1038/srep43728 PMC5347119

[37] Kwan B. C., Kronenberg F., Beddhu S., and Cheung A. K. Lipoprotein metabolism and lipid management in chronic kidney disease. J. Am. Soc. Nephrol. 18: 1246–1261, 2007; 10.1681/ASN.2006091006 17360943

[38] Liu Y., Coresh J., Eustace J. A., Longenecker J. C., Jaar B., Fink N. E., Tracy R. P., Powe N. R., and Klag M. J. 2004 Association between cholesterol level and mortality in dialysis patients: role of inflammation and malnutrition. JAMA. 291: 451–459. 14747502 10.1001/jama.291.4.451

[39] Lumlertgul D., Kantachuvesiri S., Apichaiyingyurd S., Treamtrakanpon W., Rattanasompattikul M., Gojaseni P., Thanakitcharu P., Trakarnvanich T., Poonvivatchaikarn U., and Vareesangthip K.; Impact-CKD Investigators. 2017 Prevalence of and predictive factor for abdominal aortic calcification in Thai chronic kidney disease patients. Ther. Apher. Dial. 21: 611–619. 28976071 10.1111/1744-9987.12581

[40] Hu X., Shang J., Yuan W., Zhang S., Jiang Y., Zhao B., Duan Y., Xiao J., and Zhao Z. Effects of paricalcitol on cardiovascular outcomes and renal function in patients with chronic kidney disease: a meta-analysis. Herz. Epub ahead of print. August 23, 2017; doi:10.1007/s00059-017-4605-y. 10.1007/s00059-017-4605-y 28835982

[41] Kaysen G. A., Grimes B., Dalrymple L. S., Chertow G. M., Ishida J. H., Delgado C., Segal M., Chiang J., Dwyer T., and Johansen K. L. 2018 Associations of lipoproteins with cardiovascular and infection-related outcomes in patients receiving hemodialysis. J. Clin. Lipidol. 12: 481–487.e14. 29361496 10.1016/j.jacl.2017.12.007 PMC5880742

[42] Shor R., Wainstein J., Oz D., Boaz Z., Matas M., Fux A., and Halabe A. 2007 Low serum LDL cholesterol levels and the risk of fever, sepsis, and malignancy. Ann. Clin. Lab. Sci. 37: 343–348. 18000291

[43] Vyroubal P., Chiarla C., Giovannini I., Hyspler R., Ticha A., Hrnciarikova D., and Zadak Z. 2008 Hypocholesterolemia in clinically serious conditions–review. Biomed. Pap. Med. Fac. Univ. Palacky Olomouc Czech Repub. 152: 181–189. 19219206 10.5507/bp.2008.029

[44] Liao K. P., Playford M. P., Frits M., Coblyn J. S., Iannaccone C., Weinblatt M. E., Shadick N. S., and Mehta N. N. 2015 The association between reduction in inflammation and changes in lipoprotein levels and HDL cholesterol efflux capacity in rheumatoid arthritis. J. Am. Heart Assoc. 4: e001588. 25637346 10.1161/JAHA.114.001588 PMC4345878

[45] Chien J. Y., Jerng J. S., Yu C. J., and Yang P. C. 2005 Low serum level of high-density lipoprotein cholesterol is a poor prognostic factor for severe sepsis. Crit. Care Med. 33: 1688–1693. 16096442 10.1097/01.ccm.0000171183.79525.6b

[46] Feingold K. R., Funk J. L., Moser A. H., Shigenaga J. K., Rapp J. H., and Grunfeld C. 1995 Role for circulating lipoproteins in protection from endotoxin toxicity. Infect. Immun. 63: 2041–2046. 7729918 10.1128/iai.63.5.2041-2046.1995 PMC173262

[47] Bhakdi S., Tranum-Jensen J., Utermann G., and Füssle R. 1983 Binding and partial inactivation of *Staphylococcus aureus* alpha-toxin by human plasma low density lipoprotein. J. Biol. Chem. 258: 5899–5904. 6853557

[48] Harris H. W., Grunfeld C., Feingold K. R., and Rapp J. H. 1990 Human VLDL and chylomicrons can protect against endotoxin induced death in mice. J. Clin. Invest. 86: 696–702. 2394827 10.1172/JCI114765 PMC296783

[49] Mathison J., Tobias P., Wolfson E., and Ulevitch R. 1991 Regulatory mechanisms of host responsiveness to endotoxin (lipopolysaccharide). Pathobiology. 59: 185–188. 1883513 10.1159/000163641

[50] Munford R. S., Hall C. L., Lipton J. M., and Dietschy J. M. 1982 Biological activity, lipoprotein binding behavior and in vivo disposition of extracted and native forms of *Salmonella typhimurium* lipopolysaccharides. J. Clin. Invest. 70: 877–888. 6749904 10.1172/JCI110684 PMC370296

[51] Ulevitch R. J., and Johnston A. R. 1978 The modification of the biophysical and endotoxic properties of bacterial lipopolysaccharide by serum. J. Clin. Invest. 62: 1313–1324. 372234 10.1172/JCI109252 PMC371897

[52] Ulevitch R. J., Johnston A. R., and Weinstein D. B. 1979 New function for high density lipoproteins: their participation in intravascular reactions of bacterial lipopolysaccharides. J. Clin. Invest. 64: 1516–1524. 227936 10.1172/JCI109610 PMC371301

[53] Van Lenten B. J., Fogelman A. M., Haberland M. E., and Edwards P. A. 1986 The role of lipoproteins and receptor-mediated endocytosis in the transport of bacterial lipopolysaccharide. Proc. Natl. Acad. Sci. USA. 83: 2704–2708. 3517876 10.1073/pnas.83.8.2704 PMC323368

[54] Chan W., Bosch J. A., Phillips A. C., and Chin S. H. 2018 The associations of endotoxemia with systemic inflammation, endothelial activation, and cardiovascular outcome in kidney transplantation. J. Ren. Nutr. 28: 13–27. 29089280 10.1053/j.jrn.2017.06.004

[55] Madan N., and Kaysen G. A. 2018 Gut endothelial leakage of endotoxin may be the source of vascular inflammation and injury in CKD. How can this be targeted? J. Ren. Nutr. 28: 1–3. 29249294 10.1053/j.jrn.2017.11.001

[56] Lamping N., Dettmer R., Schröder N. W., Pfeil D., Hallatschek W., Burger R., and Schumann R. R. 1998 LPS-binding protein protects mice from septic shock caused by LPS or Gram-negative bacteria. J Clin Invest. 101: 2065–2071. 9593762 10.1172/JCI2338 PMC508794

[57] Levels J. H., Marquart J. A., Abraham P. R., van den Ende A. E., Molhuizen H. O., van Deventer S. J., and Meijers J. C. 2005 Lipopolysaccharide is transferred from high-density to low-density lipoproteins by lipopolysaccharide-binding protein and phospholipid transfer protein. Infect. Immun. 73: 2321–2326. 15784577 10.1128/IAI.73.4.2321-2326.2005 PMC1087464

[58] Havel R. J., and Hamilton R. L. 1988 Hepatocytic lipoprotein receptors and intracellular lipoprotein catabolism. Hepatology. 8: 1689–1604. 2847970 10.1002/hep.1840080637

[59] van Dijk P. C., Jager K. J., de Charro F., Collart F., Cornet R., Dekker F. W., Riska-Grönhagen C., Kramar R., Leivestad T., Simpson K., ; ERA-EDTA Registry. 2001 The results of a collaborative effort by the ERA-EDTA registry and six national or regional registries. Nephrol. Dial. Ttransplant. 16: 1120–1129. 10.1093/ndt/16.6.1120 11390709

[60] Touvier M., Fassier P., His M., Norat T., Chan D. S., Blacher J., Hercberg S., Galan P., Druesne-Pecollo N., and Latino-Martel P. 2015 Cholesterol and breast cancer risk: a systematic review and meta-analysis of prospective studies. Br. J. Nutr. 114: 347–357. 26173770 10.1017/S000711451500183X

[61] Zhou D. C., Yang X. H., Zhan X. L., Gu Y. H., Guo L. L., and Jin H. M. 2018 Association of lean body mass with nutritional parameters and mortality in hemodialysis patients: a long-term follow-up clinical study. Int. J. Artif. Organs. 41: 297–305. 29562797 10.1177/0391398818762355

[62] Dai L., Mukai H., Lindholm B., Heimbürger O., Barany P., Stenvinkel P., and Qureshi A. R. 2017 Clinical global assessment of nutritional status as predictor of mortality in chronic kidney disease patients. PLoS One. 12: e0186659. 29211778 10.1371/journal.pone.0186659 PMC5718431

