# Supplementary material for: Lipid levels are inversely associated with infectious and all-cause mortality: international MONDO study results
Source: J Lipid Res. 2018 Jun 12;59(8):1519–28. doi: 10.1194/jlr.P084277 (PMC6071781; doi:10.1194/jlr.P084277)
Supplement: Supplemental Data [file supp_59_8_1519__index.html]

Lipid levels are inversely associated with infectious and all-cause mortality: international MONDO study results — Supplemental Data 

# Lipid levels are inversely associated with infectious and all-cause mortality: international MONDO study results

## Supplemental Data

- [supplemental materials] (.pdf, 677 KB) - This is the additional supplemental materials for the response to reviewer 1 's comment 2
