# Supplementary material for: Lipid levels are inversely associated with infectious and all-cause mortality: international MONDO study results
Source: J Lipid Res. 2018 Jun 12;59(8):1519–28. doi: 10.1194/jlr.P084277 (PMC6071781; doi:10.1194/jlr.P084277)

Reviewer 1

Comment 2:

KM Plot by using the average value during the baseline period:

All-cause mortality

LDL-C

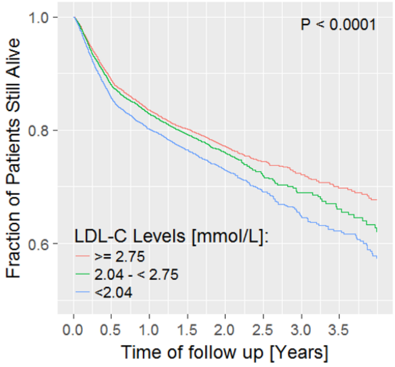

HDL-C

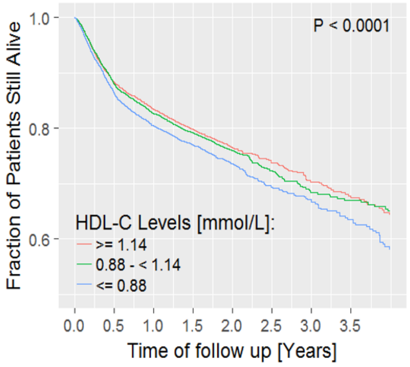

TG

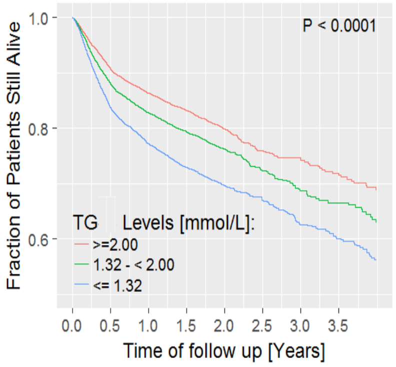

CV-related mortality

LDL-C

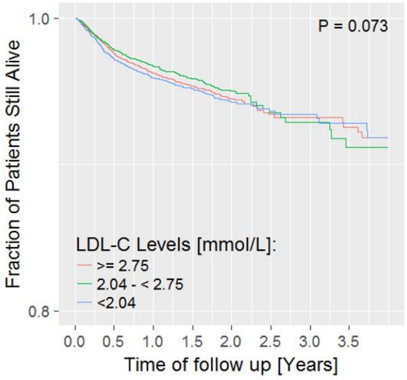

HDL-C

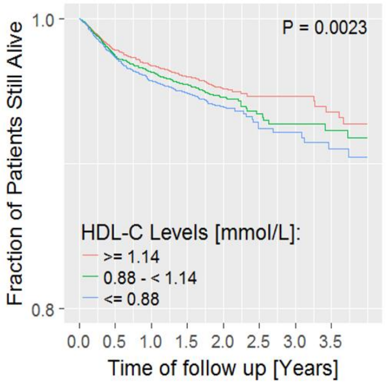

TG

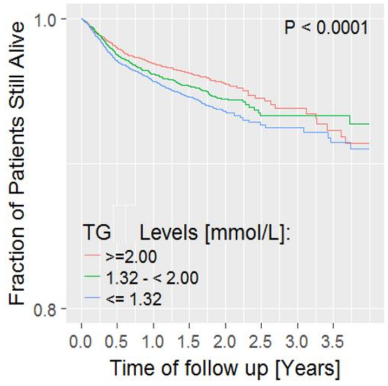

All-infectious related mortality

LDL-C

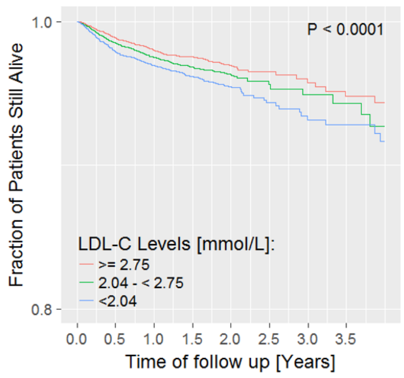

HDL-C

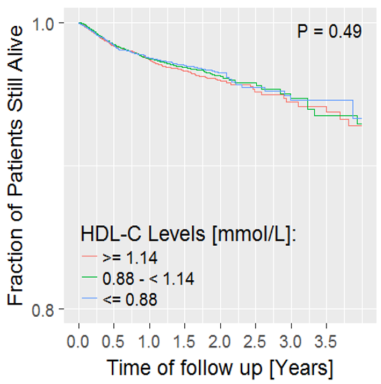

TG

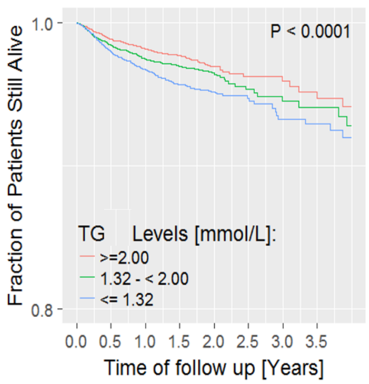

Other-causes (not infectious and CV related) mortality

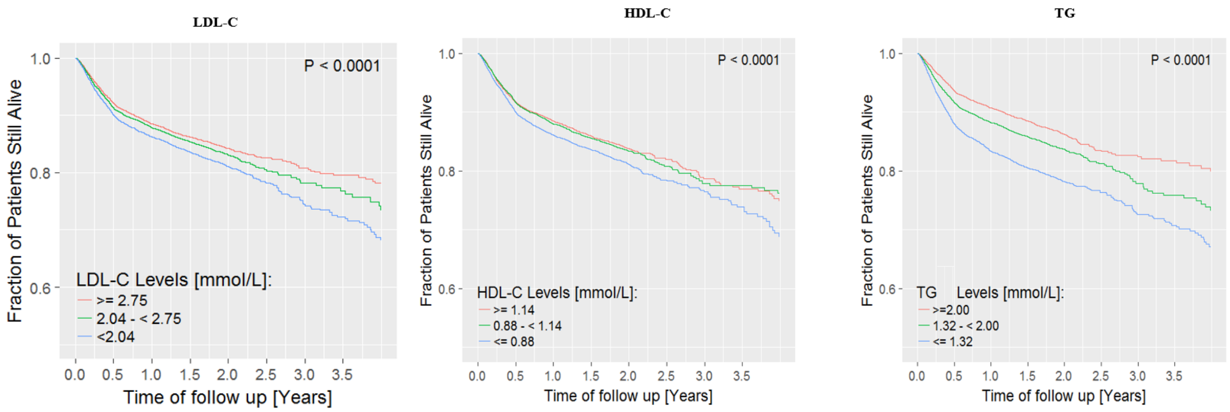

Supplement: Supplemental Data [file 10.1194_P084277_jlr.P084277-1.pdf]
